# Safety and hemostatic efficacy of fibrin pad in partial nephrectomy: Results of an open-label Phase I and a randomized, standard-of-care-controlled Phase I/II study

**DOI:** 10.1186/1471-2369-13-147

**Published:** 2012-11-08

**Authors:** Ofer Nativ, Bababhai Patel, Jessica Shen, Jonathan Batiller, Sara Horn, James C Hart

**Affiliations:** 1 Bnai Zion Medical Center, Haifa, Israel; 2 Ethicon, Inc, A Johnson and Johnson Company, P. O. Box #151, Somerville, NJ 08876-0151, USA; 3 Omrix Biopharmaceuticals Ltd, Kiryat Ono, Israel

**Keywords:** Fibrin pad, Fibrinogen, Hemostasis, Nephron-sparing surgery, Oxidized-regenerated cellulose, Polyglactin 910, Thrombin

## Abstract

**Background:**

Bleeding severity, anatomic location, tissue characteristics, and visibility are common challenges encountered while managing intraoperative bleeding, and conventional hemostatic measures (suture, ligature, and cautery) may sometimes be ineffective or impractical. While topical absorbable hemostats (TAH) are useful hemostatic adjuvants, each TAH has associated disadvantages.

**Methods:**

We evaluated the safety and hemostatic efficacy of a new advanced biologic combination product―fibrin pad―to potentially address some gaps associated with TAHs. Fibrin pad was assessed as adjunctive hemostat in open partial nephrectomy in single-center, open-label, Phase I study (N = 10), and as primary hemostat in multicenter, single-blind, randomized, standard-of-care (SOC)-controlled Phase I/II study (N = 7) in Israel. It was used to control mild-to-moderate bleeding in Phase I and also spurting arterial bleeding in Phase I/II study. Phase I study assessed safety and Phase I/II study, proportion of successes at 10 min following randomization, analyzed by Fisher exact tests at 5% significance level.

**Results:**

Phase I (N = 10): All patients completed the study. Hemostasis was achieved within 3–4 min (average = 3.1 min) of a single application in all patients. Fibrin pad was found to be safe for human use, with no product-related adverse events reported. Phase I/II (N = 7): Hemostatic success at 10 min (primary endpoint) was achieved in 3/4 patients treated with fibrin pad versus 0/3 patients treated with SOC. No clinically significant change in laboratory or coagulation parameters was recorded, except a case of post-procedural hemorrhage with fibrin pad, which was considered serious and related to the fibrin pad treatment, and required re-operation. Although Data Safety Monitoring Board authorized trial continuation, the sponsor decided against proceeding toward an indication for primary treatment of severe arterial hemorrhage as a replacement for sutures. The study was suspended after 7/30 planned subjects were enrolled.

**Conclusions:**

The first-in-man trial of fibrin pad demonstrated its safety and efficacy as an adjunctive hemostatic technique for mild-to-moderate bleeding in partial nephrectomy. The study also suggested that the product should not replace sutures or meticulous surgical techniques for the treatment of severe arterial hemorrhage.

**Trial registration:**

Phase I/II trial, NCT00598130

## Background

Nephron-sparing surgery (NSS) is currently a preferable treatment for most patients with organ-confined renal cancer. Compared to radical nephrectomy, NSS provides excellent cancer control while providing renal function preservation and in some patients, significantly better survival [1,2]. There is increasing evidence of an association between postoperative renal function and non-cancer mortality, mainly due to cardiovascular events [3,4]. The frequent use of NSS in small renal cell cancer is attributed to the increasing incidental diagnosis of renal tumors in patients undergoing abdominal ultrasound or computed tomography (CT) for various indications [5]. NSS is not yet completely accepted by urologists, primarily because of difficulty in controlling bleeding and the consequent hemodynamic instability [6,7]. Hemostasis can be achieved by conventional surgical techniques such as suture ligature, cautery, and argon beam coagulation. However, these techniques are sometimes time consuming and may prove ineffective for persistent, extensive bleeding. More recently, adjunctive hemostatic techniques using topical absorbable hemostats, e.g., oxidized regenerated cellulose, gelatin, or collagen and liquid fibrin sealants, have been employed to decrease intraoperative renal ischemic time, provide rapid hemostasis, and when surgical fields are difficult to visualize [6,8-11]. Liquid sealants serve as effective hemostatic adjuncts but may have limited use in high-volume or high-pressure bleeding [12].

Fibrin pad (OMRIX Biopharmaceuticals Ltd., Israel; Ethicon Inc., USA) was designed with the intention to potentially overcome the limitations of other adjunctive hemostatic products. It is a sterile, bio-absorbable combination product of a coating of lyophilized biologically active, human thrombin and fibrinogen. A pre-clinical study in a swine partial nephrectomy model demonstrated that direct application of fibrin pad was effective in achieving hemostasis in every animal, with no fibrin pad-related adverse reactions [13].

Here, we report the first-in-man use of a newly developed fibrin pad tested as an adjunct to conventional hemostatic techniques, and as a primary/only method of hemostasis during NSS in 2 early phase clinical studies.

## Methods

Both studies were conducted in Israel in accordance with the requirements for conduct of clinical studies (Ministry of Health, Good Clinical Practice Standard), ICH Harmonised Tripartite Guideline for Good Clinical Practice (2000), and the Declaration of Helsinki (1996). The protocols for the studies were approved by Beilinson Hospital, Rabin Medical Center (Petah Tikva) and Bnai Zion Medical Center (Haifa) ethics committees. All patients provided written informed consent before study participation.

### Fibrin pad

**Figure 1:**
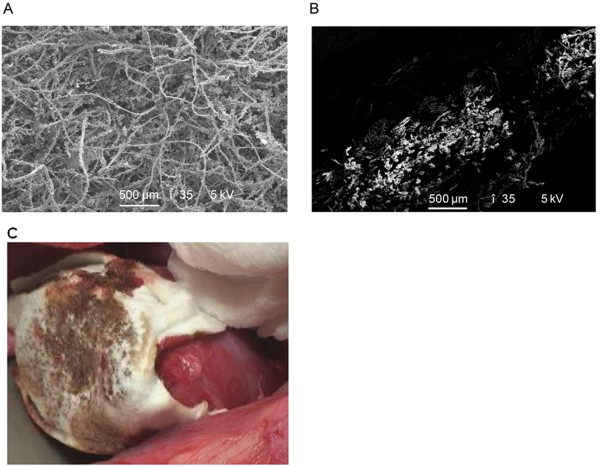
**Scanning electron micrograph (SEM) image of fibrin pad. A**) Top-view of fibrin pad, **B**) Cross section of bioactive matrix with human fibrinogen and human thrombin powders retained by polyglactin 910 fibers, and **C**) Image of the product application site during porcine partial nephrectomy.

Fibrin pad is a sterile, bio-absorbable combination product composed of lyophilized human plasma-derived fibrinogen (4.7-12.4 mg/cm^2^) and thrombin (15-45 IU/cm^2^), which is similar to EVICEL® Fibrin Sealant [Human] [14,15] coated on a flexible matrix that consists of a woven mesh of oxidized, regenerated cellulose [16] (similar to SURGICEL® Absorbable Hemostats and GYNECARE INTERCEED® Absorbable Adhesion Barrier) of plant origin, with polyglactin 910 (similar to VICRYL^TM^ suture and mesh products) filaments [13] (Figure 1A and 1B) functioning as a mechanical barrier. Fibrin pad was supplied in 10 × 10 cm^2^ units and was to be cut to fit the wound site with a margin of 1-2 cm beyond the wound. A dose equivalent to 1 unit could be left in the body. In Figure 1C, we show the application site of fibrin pad during a porcine partial nephrectomy procedure conducted in a preclinical laboratory setting.

#### Phase I study design

This single-center, open-label, prospective, non-randomized, non-controlled study assessed the safety of fibrin pad as an adjunct to conventional hemostatic procedures in patients undergoing NSS. The study included patients aged 18–75 years undergoing elective NSS for renal tumors ≤4 cm in diameter. Patients having only 1 functional kidney, undergoing additional surgical interventions, or requiring cooling of the kidney; known coagulopathy, abnormal prothrombin time, or international normalized ratio > 1.3; known intolerance to blood products or other product components; or who had recently received anticoagulants or anti-aggregates were excluded.

**Figure 2:**
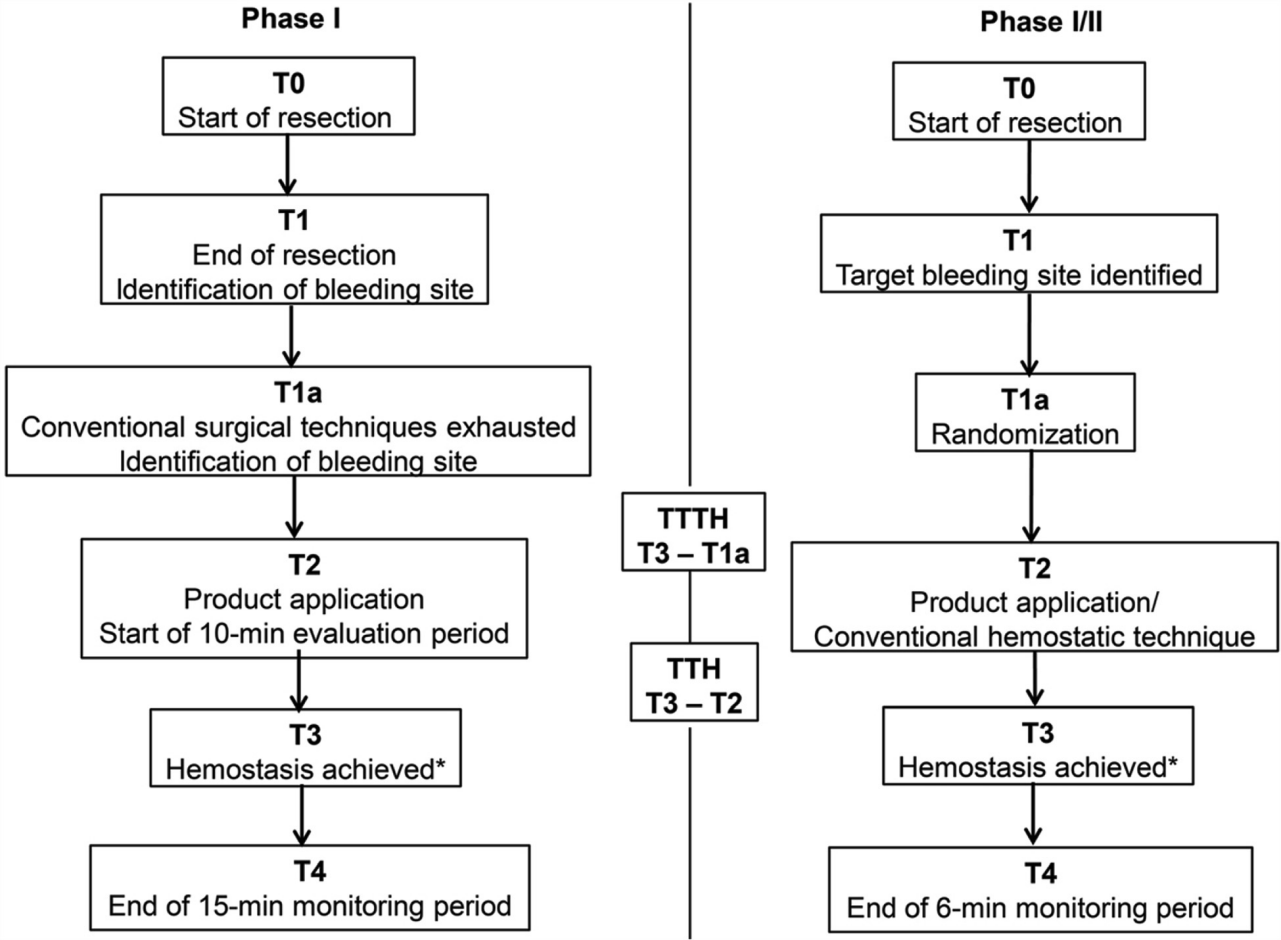
**Time-table for assessment of hemostasis.** Time points were recorded from start of kidney resection to the observation period for both the studies. Time to hemostasis (TTH) and total time for hemostasis (TTTH) were calculated from these time points. *If complete hemostasis was not achieved at 3 min, up to 2 additional applications were permitted within the 10-min evaluation period, starting immediately after the 1st application, with the total fibrin pad used not exceeding a 10 x 10 cm2 unit. If hemostasis was not achieved during the 10-min evaluation period with 3 applications of fibrin pad, the patient was recorded as treatment failure and further hemostatic measures based on physician preference were employed.

Surgery was performed according to the standard of care (SOC). Following segmental resection of the renal tissue, when conventional hemostatic measures were exhausted, fibrin pad was applied to the target bleeding site (TBS) followed by manual pressure for 3 min; bleeding status was evaluated for 10 min since application to assess the time required to achieve hemostasis (Figure 2).

CBC, coagulation parameters, volume and type of blood products transfused, and drainage fluid volume (if applicable) were recorded. Follow-up visits occurred 2, 4, and 8 weeks post-surgery. Adverse events (AEs), including bleeding-related complications, were monitored throughout the study.

The primary endpoint was evaluation of safety based on AE incidence and clinically abnormal laboratory and coagulation parameters. Secondary endpoints included time to hemostasis (TTH), time to total hemostasis (TTTH), incidence of treatment failure and re-bleeding, and surgeon’s evaluation of fibrin pad use. All statistical analyses were performed using SAS version 9.1 (SAS Institute, Cary, NC, USA).

#### Phase I/II study design

This was a prospective, multicenter, randomized, single-blind, SOC-controlled study, which planned to recruit 30 subjects in 2 cohorts. The first cohort of 10 eligible subjects was to be randomized in a 1:1 ratio to the fibrin pad or SOC (electrosurgery, suture, absorbable hemostat, and SURGICEL®) group. Inclusion/exclusion criteria were similar to those of the Phase I study, except that the patients were 18–65 years old and were excluded if the intraoperative blood collection by suction exceeded 500 mL (excluding irrigation liquids), sustained systolic pressure was <80 mmHg, sustained heart rate was ≥130 beats per minute, or sustained oxygen saturation (SaO_2_) was <90%. Screening criteria and baseline assessments performed 24 h prior to the procedure were similar to those of the Phase I study.

Randomization occurred once all intraoperative inclusion criteria were met, and an appropriate TBS was identified following tumor excision. Treatment allocation happened through an interactive voice response system. Fibrin pad was applied to the TBS after removal of excess blood or fluids, and hemostasis was assessed for 10 min followed by a 6-min observation period (Figure 2). In subjects randomized to the control arm, bleeding was controlled by conventional surgical techniques and hemostasis was assessed at similar time points as in the fibrin pad group. In cases where vascular occlusion was used, fibrin pad was applied simultaneously with the opening of the clamp. Arterial clamping was employed in Phase I study but not during active hemostat application in Phase II. Intraoperative measurements and evaluation of AEs were similar to those of the Phase I study. Follow-up visits occurred at 2 weeks and 1 month.

The primary efficacy endpoint was the proportion of successes at 10 min following randomization (defined as complete hemostasis within 10 min and no further bleeding during the subsequent 6-min observation period). Secondary efficacy endpoints included proportion of successes at 5 min following randomization, laboratory assessments, safety, and parameters similar to the secondary endpoints of the Phase I study.

The 2 treatment groups were compared for the proportion of successes at 10 min and 5 min using Fisher exact tests at a 5% significance level. For continuous variables such as TTH, the 2 treatment groups were compared using an analysis of variance (ANOVA) with study center as a factor. For non-parametric tests, the Wilcoxon rank-sum procedure with a continuity correction was used.

## Results

### Phase I study

**Figure 3:**
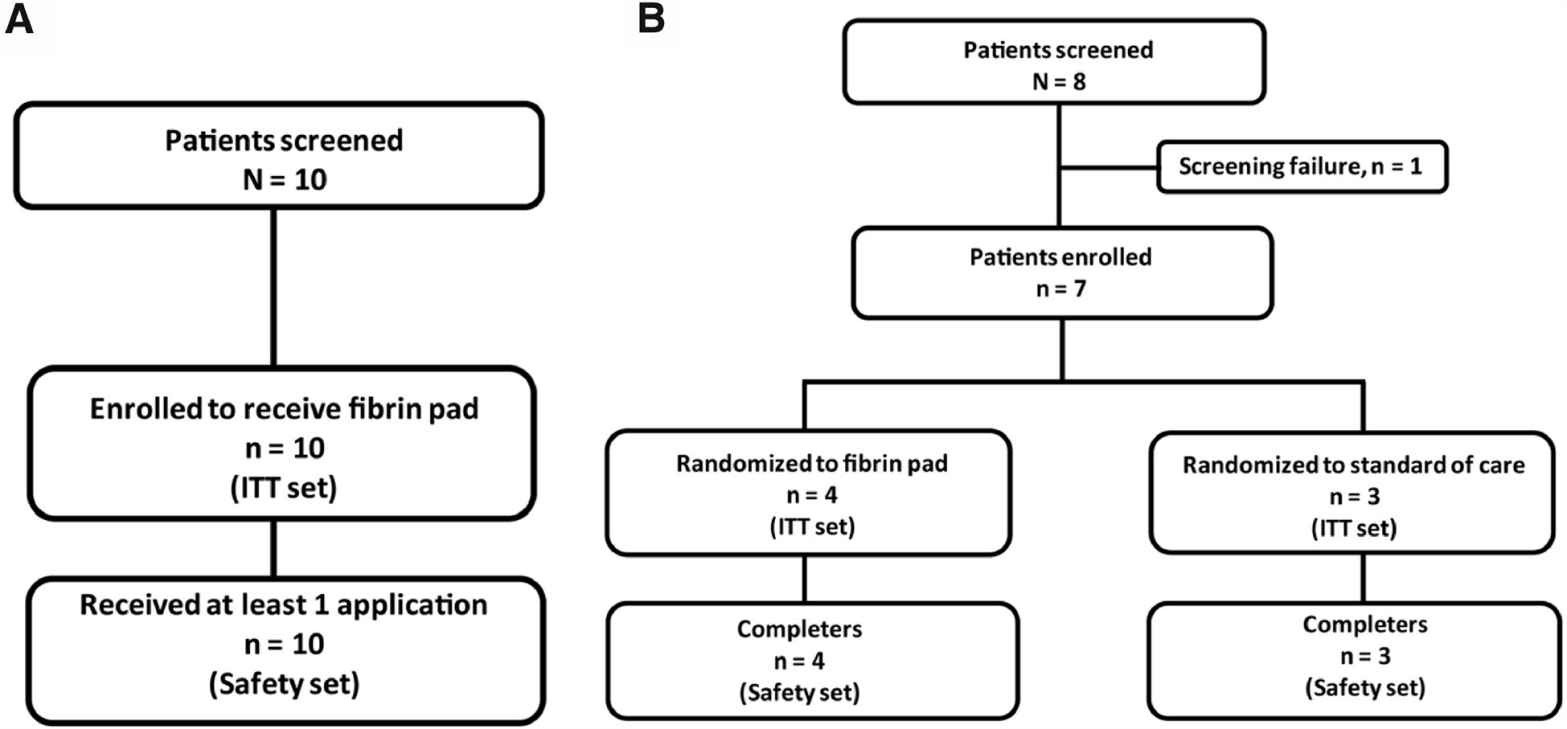
Patient disposition of Phase I (Panel A) and Phase II (Panel B) studies.

**Figure 4:**
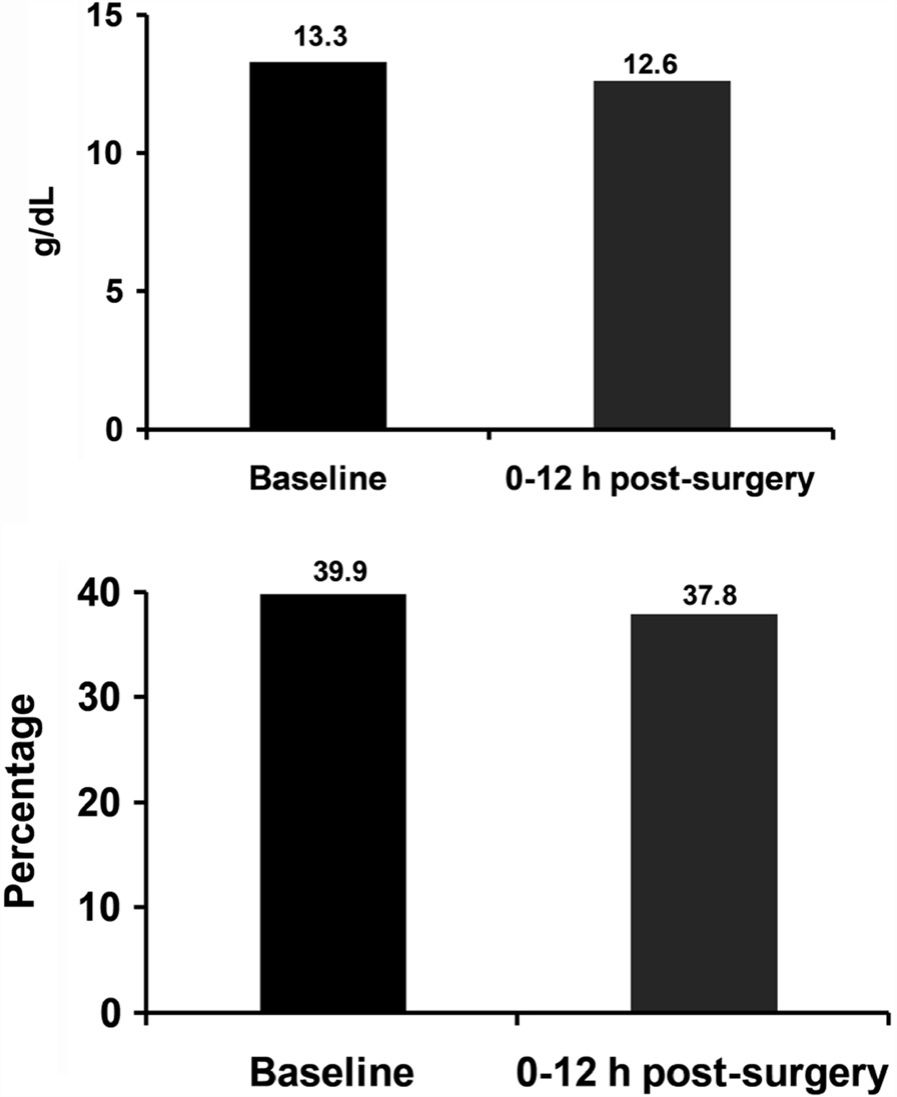
**Changes in hemoglobin and hematocrit levels from baseline to 12 h post-surgery in the Phase I open-label study.** There was a mean drop of 0.86 g/dL in the hemoglobin level and 2.3% in the hematocrit level over this period.

All 10 patients (mean age [SD], 64.5 [8.87] years) enrolled between December 2006 to August 2007 completed the study (Figure 3). Table 1 shows their demographic data. Each patient received a single application of fibrin pad; the mean estimated pad size used was 30.5 cm^2^ (range, 9–64 cm^2^). A total of 19 treatment-emergent AEs were reported, with 9 of 10 patients experiencing at least 1 AE (Table 2A). There were no deaths, serious adverse events (SAEs), or treatment-related AEs. All treatment-emergent AEs were rated as mild, except for 1 case of hypokalemia that was moderate in intensity. Deviations in coagulation and hematology parameters were transient, not clinically significant, and expected due to the nature of the procedure. Figure 4 shows the decrease in hemoglobin (Hb) and hematocrit (Hct) levels from baseline to 12 h post-surgery. The TTH was 3 min in 9 of 10 patients (90%) and 4 min in 1 patient. TTTH was 5–10 min in the 10 patients. No treatment failure or re-bleeding was reported, and no patient required further surgery during the 8-week follow-up period. The Ease of Use Questionnaire, completed by the 2 surgeons who performed the surgery, rated the product as relatively easy to use and assessed the product as able to achieve hemostasis rapidly and safely.

**Table 1:** Demographics and baseline characteristics

**Characteristic**	**Phase I**	**Phase I/II**	
	**Fibrin pad N = 10**	**Fibrin pad N = 4**	**Standard of care N = 3**
Age (years), mean (SD)	64.5 (8.87)	56.0 (7.87)	59.0 (14.11)
Weight (kg), mean (SD)	75.1 (10.41)	84.3 (14.15)	78.3 (18.15)
Height (cm),* mean (SD)	164.7 (10.95)	166.3 (11.44)	170.7 (8.08)
Gender, n (%)			
Male	8 (80)	3 (75.0)	2 (66.7)
Female	2 (20)	1 (25.0)	1 (33.3)
Race, n (%)			
Caucasian	10 (100)	4 (100)	3 (100)

* n = 7.

**Table 2:** Summary of adverse events

**(A) Phase I study**		
	**N = 10**	
Any AE, n (%)	9 (90.0)	
SAE, n (%)	0 (0.0)	
AEs related to investigational product, n (%)	0 (0.0)	
Most common AEs, n (%)		
Nausea	2 (20.0)	
Pyrexia	6 (60.0)	
Procedural site reaction	2 (20.0)	
**(B) Phase I/II study**		
	**Fibrin pad (N = 4)**	**Standard of care (N = 3)**
Total number of AEs	2	2
Incidence of AEs, n (%)	1 (25.0)	1 (33.3)
Incidence of SAEs, n (%)	1 (25.0)	0 (0.0)
AEs related to investigational product, n (%)		
Post-procedural hemorrhage	1 (25.0)	0 (0.0)
Most common AEs, n (%)		
Epididymitis	1 (25.0)	0 (0.0)
Atelectasis	0 (0.0)	1 (33.3)
Postoperative wound infection	0 (0.0)	1 (33.3)

*AE*, adverse event; *SAE*, serious adverse event.

### Phase II study

The study population was evenly distributed between the fibrin pad (n = 4) and SOC (n = 3) groups, with the mean (SD) age of the subjects being 57.3 (10.00) years. All 7 subjects completed the study between May–October 2008 (Figure 3). Table 1 shows the demographic data. The study was suspended according to the protocol stopping rules owing to an event of post-procedural hemorrhage from the TBS. This event was assessed by the study data and safety monitoring board (DSMB), who recommended study continuation. However, the sponsor made the decision to not pursue the use of fibrin pad as the primary and sole treatment of severe arterial hemorrhage that would require mechanical ligation.

#### Primary efficacy endpoint

Hemostatic success at 10 min was achieved in 3 of 4 patients (75%) in the fibrin pad group versus none of the 3 patients (0%) in the SOC group. However, this result was not statistically significant given the small number of observations.

#### Secondary efficacy endpoints

Hemostatic success at 5 min was not achieved in any patient in either group. The median TTH and TTTH were shorter in the fibrin pad group than in the SOC group, but neither difference was statistically significant (Table 3). All subjects in the fibrin pad group required 2 applications because hemostasis was not achieved in 3 patients within 3 min of application and there was an instance of re-bleeding in 1 patient during the 6-min observation period. All 3 patients in the SOC group were listed as treatment failures. The decrease in Hb and Hct levels from baseline to the day of discharge was lower in the fibrin pad group than in the SOC group. Changes in the hematology and coagulation laboratory parameters were not considered clinically significant, except for those in the patient who experienced post-procedural hemorrhage.

**Table 3:** Time to hemostasis

	**Phase I**^**†**^	**Phase I/II**^**†**^		
	**Fibrin pad N = 10**	**Fibrin pad N = 4**	**Standard of care N = 3**	*P *value*
**Time to hemostasis (min)**				
Mean (SD)	3.1 (0.31)	15.0 (13.41)	21.0 (7.94)	
Median (range)	3 (3–4)	8.4 (8–35)	18.0 (15–30)	0.41
**Total time to hemostasis (min)**				
Mean (SD)	6.2 (1.54)	16.0 (13.00)	21.3 (8.50)	
Median (range)	6 (5–10)	9.0 (9–36)	18.0 (15–31)	0.41

^*^Fibrin pad was used as an adjunct in Phase I and as the primary treatment in Phase I/II.

^**†**^Wilcoxon 2-sample test.

#### Safety

A total of 4 AEs were reported in 2 subjects in each group during the study period (Table 2). Three events were non-serious, mild in intensity, and considered unrelated to the study treatment, whereas 1 event, an episode of post-procedural hemorrhage in a subject in the fibrin pad group, was considered serious, severe in intensity, and related to the study treatment. Bleeding was observed in the drain 2 h after surgery, and the patient was hemodynamically instable. This episode required the subject to be re-operated, and the source of bleeding was the resection area beneath the fibrin pad. The fibrin pad was removed, and hemostasis was achieved using a standard surgical technique. This patient was administered a total of 10 units of blood in the postoperative period and was discharged from the hospital without sequelae.

## Discussion

NSS is performed by many urologists endoscopically, and it has emerged as an alternative to radical nephrectomy for removal of small (≤4 cm diameter) as well as larger renal masses, since cancer-specific survival outcomes are similar for both approaches, even in young patients [17,18]. However, achieving rapid and effective hemostasis in NSS can be challenging. Fibrin pad was designed to provide surgeons an alternative adjunct to hemostatic techniques for achieving rapid control of mild, moderate, and severe bleeding in a variety of tissue types. In the current Phase I study, complete hemostasis was achieved in all patients within 3–4 min of a single application of the fibrin pad, without the occurrence of re-bleeding. This has immense clinical significance as it has been reported that each additional minute of warm ischemia following partial nephrectomy is associated with a 5% increase in the odds of developing acute renal failure, 6% increase in the odds of a glomerular filtration rate of <15 mL/min per 1.73 m^2^ in the postoperative period, and a 6% increase in the risk of new-onset stage IV chronic kidney disease during follow-up [19]. Technologies similar to the fibrin pad have been demonstrated to be effective in sealing air leakage in the lung [20] and for achieving hemostasis in the kidney [14] and liver [21]. TachoSil^TM^, an equine collagen patch coated on one side with human fibrinogen and thrombin, has been used in clinical trials; however, animal collagen could be a concern in allergic patients [22-24].

In the Phase II study, where fibrin pad was used as the primary and sole hemostat, the upper limit of product performance was tested by assessing its efficacy in controlling severe arterial bleeding from the highly vascular renal parenchyma. Although hemostasis was achieved, all patients in the fibrin pad group required reapplication of the product. This may be attributed to the learning curve of surgeons and also to the fact that the fibrin pad requires direct and complete product-to-tissue surface apposition with persistent manual compression to activate the biologic components and develop tissue adherence, which may have been challenging given the irregular wound geometry. Intraoperative treatment failure was observed in all 3 patients in the SOC group, where vascular clamping was used. Since no vascular clamping was required in the fibrin pad group, the possibility of ischemia is minimal.

Fibrin pad builds on the EVICEL® liquid fibrin sealant technology consisting of virus-inactivated, human plasma-derived thrombin and fibrinogen [15,25]. The 2 components are mixed during application and upon combination, mimic the final step in the blood coagulation pathway to form a stable clot [11,26]. Furthermore, the biological components of the fibrin pad are free of tranexamic acid and aprotinin, which have been associated with neurological AEs and hypersensitivity reactions, respectively [27,28]. The fibrin sealant may be rendered ineffective if it is removed from the TBS due to adhesive or cohesive failure, particularly in severe bleeding [12,24,29,30]. In a prospective, randomized controlled trial that compared the safety and hemostatic effectiveness of a fibrin sealant (EVICEL®; 75 patients) to manual compression (72 patients) during vascular surgery, Chalmers et al. demonstrated that a higher percentage of patients who received the fibrin sealant achieved hemostasis at 4 min as compared to those who received manual compression (85% vs. 39%; *P* < 0.001) [15], showing that biological components present in the fibrin pad are effective in achieving hemostasis.

While fibrin sealants have proven efficacy in intraoperative hemostasis, Cornum et al. evaluated the ability of an absorbable fibrin adhesive bandage (AFAB), a prototype product comprising lyophilized fibrinogen and thrombin on a VICRYL mesh backing, to seal the collecting system and control bleeding after partial nephrectomy in growing pigs [8]. Compared to conventional management, the use of AFAB resulted in significantly less bleeding (357 mL vs. 13 mL with AFAB; *P* < 0.001), shorter operative and ischemic times, a stable clot, and healing that is at least as successful as conventional treatments. A case series on the use of fibrin sealant applied over an absorbable hemostat, SURGICEL® after argon beam coagulation in 15 patients undergoing laparoscopic wedge resection of small renal lesions reported that hemostasis was achieved in all patients, and no blood transfusion was required [16]. These studies demonstrated that a combination of biological components on a bio-absorbable pad, as used in the current study, was effective for hemostasis.

Furthermore, a pre-clinical study in a severe renal bleeding swine model demonstrated that fibrin pad was as effective as conventional therapy in achieving durable hemostasis, and no case of re-bleeding or systemic/local adverse response was observed during the 8-week follow-up period [13].

## Conclusions

This paper reports the first-in-man use of fibrin pad, a potential life-saving technology, as an adjunctive hemostat. All patients in the Phase I study demonstrated complete and rapid hemostasis with the fibrin pad without any safety concerns. The Phase II study tested the upper limit of product performance and suggested that the product should be used as an adjunct to hemostats and should not replace suture ligation for severe arterial hemorrhage. Subsequent clinical trials will be needed to determine the product’s clinical utility and safety profile for varying degrees of bleeding and across a spectrum of tissue types and surgical situations.

## Abbreviations

AE: Adverse events; AFAB: Absorbable fibrin adhesive bandage; ANOVA: Analysis of variance; CBC: Complete blood count; CT: Computed tomography; DSMB: Data and Safety Monitoring Board; Hb: Hemoglobin; HCT: Hematocrit; TAH: Topical absorbable hemostats; NSS: Nephron-sparing surgery; SAE: Serious adverse event; SaO_2_: Sustained oxygen saturation; SOC: Standard of care; TBS: Target bleeding site; TTH: Time to hemostasis; TTTH: Time to total hemostasis.

## Competing interests

BP, JS, JB, SH and JH are all employees of Ethicon Inc., Johnson & Johnson. ON has no competing interest to declare.

## Authors’ contributions

JS and JH contributed to the conception and design. JB, BP, SH, and ON contributed to acquisition of data. BP, JS, JH, JB and ON carried out analysis and interpretation of data. BP carried out the statistical analysis. All authors contributed to the drafting and critical revision of the manuscript. JH obtained funding, JB provided administrative, technical, or material support, and SH supervised the study. All authors read and approved the final manuscript.

## Pre-publication history

The pre-publication history for this paper can be accessed here:

http://www.biomedcentral.com/1471-2369/13/147/prepub
